# Case report: Thoracic endovascular aortic repair using a non-touch exclusion technique with a custom-made device for the treatment of a large patent ductus arteriosus

**DOI:** 10.3389/fcvm.2023.1218158

**Published:** 2023-08-17

**Authors:** Yaser Jenab, Hossein Salehi Omran, Kaveh Hosseini, Saeed Tofighi, Homa Ghaderian, Ismail Ates

**Affiliations:** ^1^ Tehran Heart Center, Cardiovascular Diseases Research Institute, Tehran University of Medical Sciences, Tehran, Iran; ^2^ Cardiac Primary Prevention Research Center, Cardiovascular Diseases Research Institute, Tehran University of Medical Sciences, Tehran, Iran; ^3^ Department of Cardiology, Faculty of Medicine, Bahcesehir University, Istanbul, Turkey

**Keywords:** thoracic endovascular aortic repair, patent ductus arteriosus, elderly PDA, non-touch exclusion technique, custom-made device

## Abstract

Patent ductus arteriosus (PDA) is a common congenital heart disease affecting roughly one in every 2,000 term births. Although most of the patients are diagnosed and treated during childhood, few cases may persist into adulthood. We presented a 27-year-old male patient with a 20.2 mm diameter PDA who was referred to our hospital with progressive fatigue and exertional dyspnea. Given the potential complications, usual techniques such as coil occlusion and duct occluders were deemed inappropriate for this patient. Thoracic endovascular aortic repair (TEVAR) using a non-touch exclusion technique was successfully performed for this patient. The patient was discharged with no major post-surgical complications. TEVAR could be a new, safe, and effective alternative treatment to other transcatheter procedures for complicated PDAs in some patients.

## Introduction

Patent ductus arteriosus (PDA) is a prevalent congenital cardiovascular disorder that affects nearly one in every 2,000 term infants and accounts for 5%–10% of all cases of congenital heart disease (1). Although patients are usually identified and treated during childhood, few cases may remain intact until adulthood (2).

Currently, various surgical options are available that are effective and less invasive for closing PDA. Nonetheless, adult PDAs tend to be more complex in terms of size and shape, which can result in additional complications when using routine techniques. Consequently, selecting the most suitable cardiovascular repair technique for such patients has remained a matter of debate (3–5).

Using a non-touch exclusion technique in conjunction with thoracic endovascular aortic repair (TEVAR) may offer a viable alternative for treating PDA in high-risk complex patients (6). We present an adult patient with a large PDA treated successfully with TEVAR using a custom-made device.

## Case description

### Present illness and physical examination

A 27-year-old man complaining of exertional dyspnea functional class III, palpitation, and progressive fatigue was referred to our hospital for further evaluation. During the physical examination, a continuous murmur with systolic prominence was detected at the left second intercostal space and a loud pulmonary component of second heart sound was heard at the left sternal border second intercostal space, along with a blood pressure of 135/65 mm Hg and a heart rate of 85 beats/min. Additionally, oxygen saturation in both arms and legs was 98% and no cyanosis and clubbing were detected.

### Imaging

**Figure 1:**
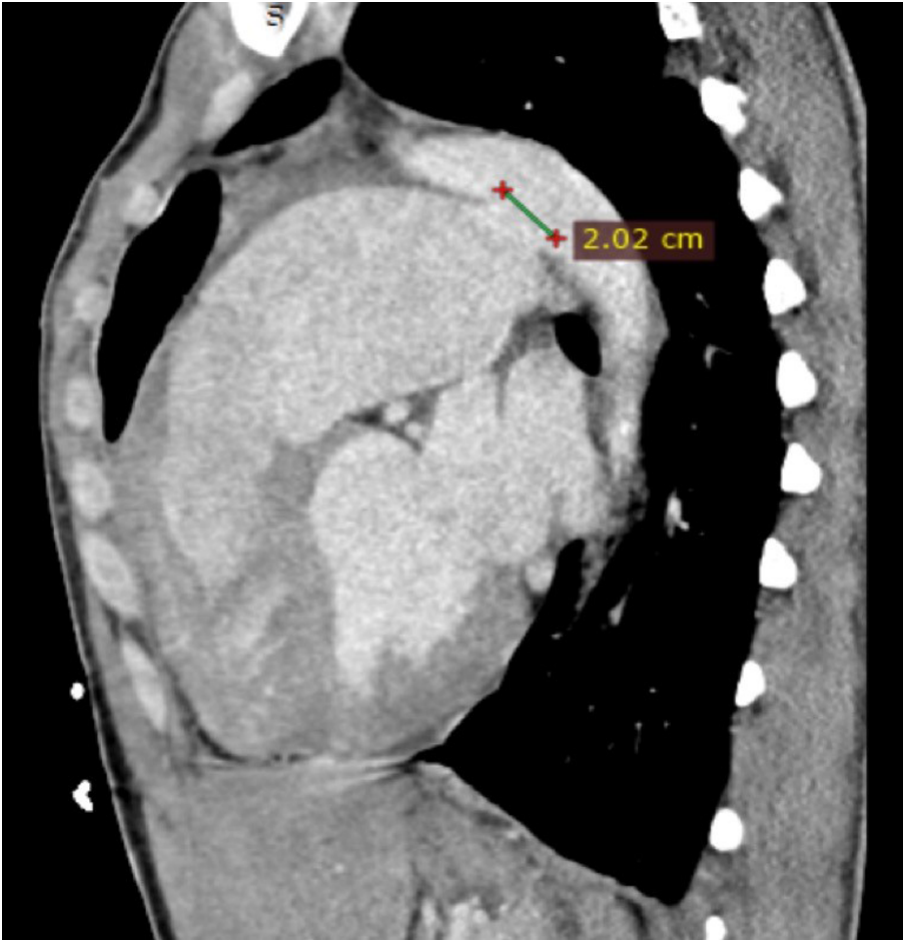
Cardiac CT in sagittal view shows a large PDA (20.2 mm) with a dilated pulmonary artery. CT, computed tomography; PDA, patent ductus arteriosus.

Transthoracic echocardiography (TTE) revealed severe left ventricular dilatation during diastole (73 mm) and an ejection fraction of 55%. Additionally, it showed that the patient has a bicuspid aortic valve with moderate aortic regurgitation (AR), as well as a mildly dilated ascending aorta (38 mm). Furthermore, the echocardiography indicated the presence of PDA, severe pulmonary valve regurgitation, and a significant enlargement of the pulmonary artery (73 mm). Following contrast-enhanced computed tomography (CT), it was found that the patient has a 20.2 mm PDA (Figure 1), which originates from the descending aorta, and 7 mm distal to the orifice of the left subclavian artery (LSA). According to the Krichenko classification, the PDA was classified as type B, which refers to a large duct with a short window-like structure at the aortic insertion (7).

### Indication for intervention

The patient had a mean pulmonary arterial pressure of 48 mmHg and a pulmonary vascular resistance of 2.1 WU during cardiac catheterization and hemodynamic assessment. Given the presenting symptoms, imaging results, and Qp/Qs ratio of 2.1, it was decided to perform a PDA closure procedure for this patient. Device closure is generally the preferred method for treating PDA, even in the presence of other concomitant cardiac lesions, due to its high success rate and low incidence of complications. However, due to the anatomical features of this patient's PDA, device closure may carry risks of potential complications such as device migration, dissection, and perforation, which renders this option inappropriate. After considering various available treatment options, the heart surgery team suggested a non-touch exclusion technique using TEVAR to reduce the risks associated with other endovascular procedures. Subsequently, we provided the patient with a comprehensive explanation of all the available options. Accordingly, the TEVAR procedure was chosen based on shared decision-making with the patient. The patient provided informed consent prior to the TEVAR procedure.

### Intervention

**Figure 2:**
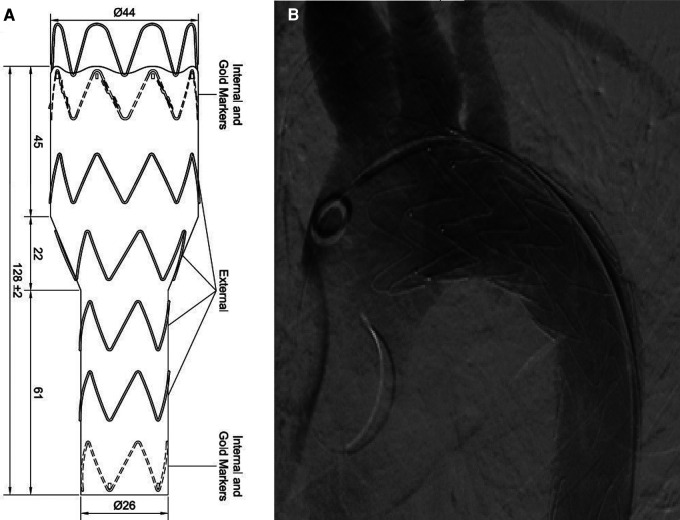
(**A**) Custom-made device. (**B**) Aortography after TEVAR revealed complete closure of PDA without any endoleaks. PDA, patent ductus arteriosus; TEVAR, thoracic endovascular aortic repair.

**Figure 3:**
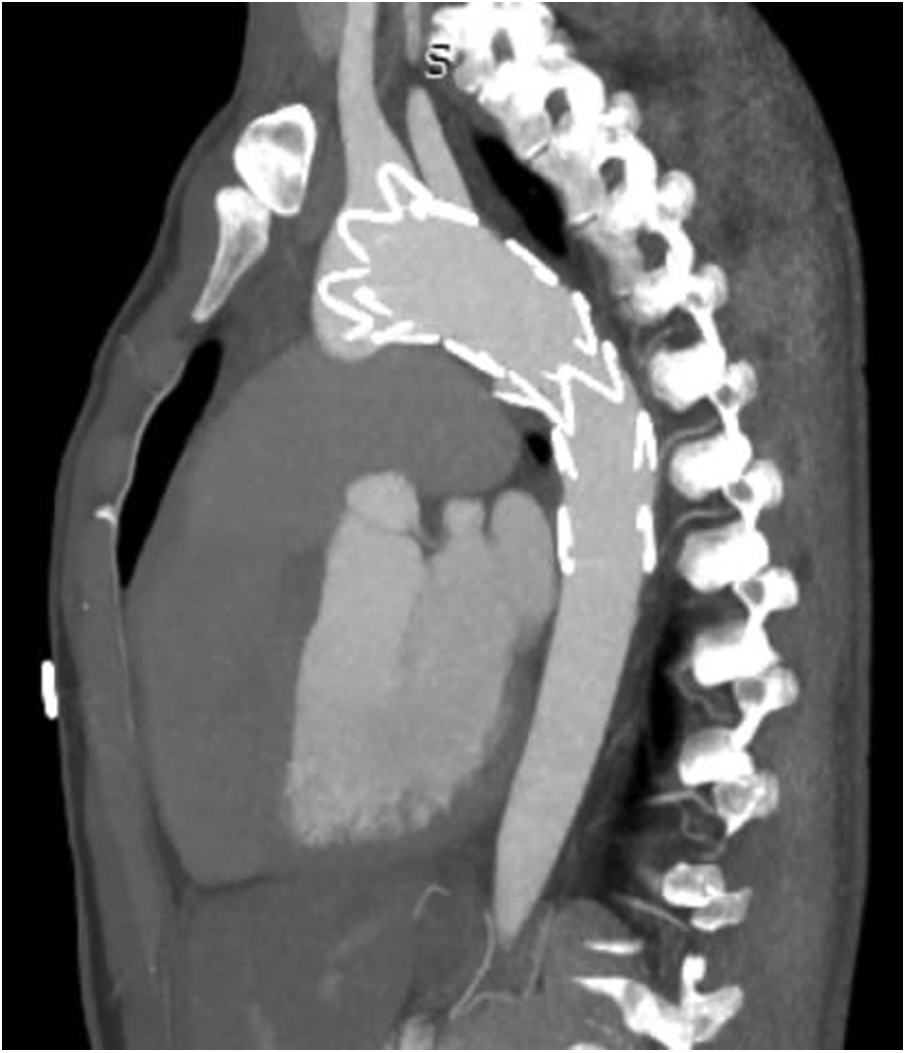
CT angiography after one month showed no endoleak with complete occlusion of PDA shunt. CT, computed tomography; PDA, patent ductus arteriosus.

TEVAR was performed while the patient was under conscious sedation, using the pre-close technique with 2 Proglide devices. Custom-made Zenith Alpha Thoracic Endovascular Graft (44 26 128 mm, Cook Medical, Bloomington, IN, USA) was inserted through the right common iliac artery. The patient underwent a zone 2 deployment of a TEVAR graft with the coverage of the LSA. Echocardiography and aortography after TEVAR showed complete closure of PDA without any endoleaks (Figure 2). The post-surgical period was uneventful, and the patient was discharged two days after the procedure with no major complications, taking interpulmonary hypertensive drugs. One month after TEVAR, CT angiography was performed that revealed complete occlusion of PDA with no endoleaks (Figure 3). Due to residual pulmonary hypertension, echocardiography is being performed annually. A detailed, comprehensive timeline of the patient's clinical course is provided in Table 1.

**Table 1:** Comprehensive timeline of the patient's clinical course in detail.

11/6/2021	The patient was referred to the congenital heart diseases clinic complaining of progressive fatigue and exertional dyspnea and underwent an echocardiography subsequently.
1/9/2022	Right heart catheterization with hemodynamic assessment were done.
1/10/2022	CT angiography was performed to evaluate the size and location of the PDA and descending aorta.
2/26/2023	The patient underwent TEVAR for PDA closure.
2/28/2023	The patient was discharged with no major complications.
3/28/2023	CT angiography performed one month after TEVAR revealed complete occlusion of PDA with no endoleaks.

CT, computed tomography; PDA, patent ductus arteriosus, TEVAR, thoracic endovascular aortic repair.

## Discussion

We presented a 27-year-old patient with a large PDA complaining of exertional dyspnea and progressive fatigue who was treated successfully with TEVAR using a custom-made device.

PDA is a prevalent congenital heart disease with an estimated incidence rate of one in every 2,000 term births (1). While most patients are typically diagnosed and treated during childhood, some cases may persist into adulthood (2). Adult PDAs tend to be more complex in terms of their shape, size, and degree of calcification compared to those diagnosed in childhood. Therefore, routine surgical procedures utilized for children may not be suitable for adult patients and leads to a controversy regarding the optimal surgical approach in these patients (3–5).

Surgical closure and percutaneous catheterization are the two main treatment options for PDA. Generally, percutaneous catheterization technologies for PDA closure are considered the most commonly used method because they are more effective and less invasive. However, endovascular intervention may not be a feasible option for complex PDAs because of severe complications. As a result, surgical closure has remained the preferred method for adults with complicated PDAs until now (8).

Several cases of successful PDA closure using the Amplatzer septal occluder have been reported in adult patients. However, this alternative option has not been approved by the Food and Drug Administration for large PDAs, and results have not been satisfactory in some case series. Additionally, there are some potential disadvantages, such as residual shunting in PDAs with some anatomical features, which may make this option inappropriate for certain patients (9).

Non-touch exclusion technique with TEVAR presents a less invasive alternative to surgical closure and may be a viable option for treating PDA in high-risk complex patients (6). Few studies have been conducted on this alternative procedure. In a case series published by Lai et al. (10), four patients who had a large PDA along with pulmonary arterial hypertension were successfully treated with TEVAR in China. Follow-up was conducted for all participants for 3 to 18 months. A TTE performed one month after surgery showed a significant decrease in pulmonary arterial pressure and left ventricle end-diastolic diameter demonstrating the success of the surgery.

Yamabe et al. (11) presented a 52-year-old patient in Japan who was referred with a 7 mm PDA. Other transcatheter procedures were deemed unsuitable for this patient due to potential complications. Consequently, an endovascular aortic repair was performed successfully, closing the PDA with a talent stent prosthesis. The post-operative follow-up period was uneventful.

In a case presented by Kato et al. (12), an adult patient with a 9.7 mm PDA underwent TEVAR. PDA was closed, and the patient was discharged with no adverse events. Similarly, in another article by Orimoto et al. (13), a woman was admitted complaining of heart failure manifestations. Echocardiography showed a PDA with 14 mm diameter that TEVAR was performed for its closure. Completion angiography and post-TEVAR CT illustrated the closure of PDA with no endoleaks.

Endo et al. (14) presented a 66-year-old man who was admitted for the treatment of a large aneurysm and type B aortic dissection as evidenced by CT angiography. Echocardiography revealed a PDA, which was closed by TEVAR through the ascending aorta. The post-operative period was uneventful with no adverse events reported.

In another study by Kim et al. (15), an adult patient in South Korea complaining of exertional dyspnea and a continuous cardiac murmur found in her physical examination was admitted to the hospital. TTE demonstrated the existence of a PDA that was treated using a non-touch exclusion technique with TEVAR without any complication reported during the follow-up period. The post-operative angiography showed closure of PDA without endoleak.

Seguchi et al. (16) presented an adult patient in Japan with PDA, AR, and coarctation of the aorta (CoA) who underwent a two-stage surgery. The first stage involved the TEVAR procedure to treat CoA and PDA with the stent graft deployment at a diameter of 31 mm after balloon dilation. A few days later, an open surgery was performed for the treatment of AR, which involved aortic valve replacement through a median sternotomy. No adverse events were reported during discharge.

In another article by Freeman et al. (17), a 31-year-old man complaining of heart failure manifestations was diagnosed with a 2.7 cm PDA. Due to the complexity of the PDA and the inability of an occlusive device to close it, other treatment options were considered. Ultimately, a zone 2 deployment of a TEVAR graft was performed for this patient, who was discharged without experiencing adverse events.

TEVAR offers several advantages over other routine techniques; at first, there is no need for catheter manipulation during this procedure. This means that unfavorable procedure-related complications such as perforation and aortic dissection are greatly reduced. Furthermore, because TEVAR devices are mainly used for treating thoracic aortic aneurysms, TEVAR can be a safe alternative for PDA closure in cases with accompanying aortic aneurysm (18).

On the other hand, there are some disadvantages. Firstly, the TEVAR procedure has its own potential complications including endoleaks, endograft collapse, and vascular access-related adverse events such as arterial rupture, perforation or dissection which can lead to retroperitoneal hemorrhage and lower limb ischemia requiring the prompt implementation of necessary measures (19). Secondly, TEVAR necessitates a relatively wide access vessel due to the larger diameter of its delivery system compared to some transcatheter procedures (20).

In addition, during a TEVAR procedure, it might be necessary to cover the LSA. Since most chronic LSA occlusions are asymptomatic, surgeons typically adopt an expectant approach following endograft occlusion of the LSA during TEVAR. This approach may lead to rare but emergent ischemic or neurologic complications related to LSA coverage which may require considering elective revascularization (21).

Furthermore, there is a controversial debate between TEVAR and other alternative procedures in terms of financial aspects. Although several studies have shown that TEVARs generally have similar or lower in-hospital charges compared to open procedures, other studies have demonstrated higher costs for TEVARs in the long-term follow up due to the need for more frequent chest follow-up imaging tests and reintervention measures (22–26).

## Conclusion

Adult PDAs that are recognized as more complex in terms of size, shape, and the presence of calcification can pose additional challenges when using routine surgical techniques. TEVAR may be a viable alternative to other transcatheter techniques, offering a safe and effective solution for treating complicated PDAs in some patients.

## Patient perspective

A 27-year-old male patient presented to our hospital with exertional dyspnea and progressive fatigue. After considering various available treatment options, we explained them to the patient who cooperated well and provided informed consent for the TEVAR procedure. The procedure was successfully performed with no adverse events, and the patient's symptoms were relieved. Follow-up CT angiography one month later showed complete occlusion of PDA without endoleak.

## Data Availability

The original contributions presented in the study are included in the article/Supplementary Material, further inquiries can be directed to the corresponding author.
